# *tbx2a* Is Required for Specification of Endodermal Pouches during Development of the Pharyngeal Arches

**DOI:** 10.1371/journal.pone.0077171

**Published:** 2013-10-10

**Authors:** Hang Nguyen Thi Thu, Steven Fong Haw Tien, Siau Lin Loh, Jimmy So Bok Yan, Vladimir Korzh

**Affiliations:** 1 Institute of Molecular and Cell Biology, Singapore, Singapore; 2 Department of Biological Sciences, National University of Singapore, Singapore, Singapore; 3 Department of Surgery, Yong Loo Lin School of Medicine, National University of Singapore, Singapore, Singapore; National University of Singapore, Singapore

## Abstract

Tbx2 is a member of the T-box family of transcription factors essential for embryo- and organogenesis. A deficiency in the zebrafish paralogue *tbx2a* causes abnormalities of the pharyngeal arches in a p53-independent manner. The pharyngeal arches are formed by derivatives of all three embryonic germ layers: endodermal pouches, mesenchymal condensations and neural crest cells. While *tbx2a* expression is restricted to the endodermal pouches, its function is required for the normal morphogenesis of the entire pharyngeal arches. Given the similar function of Tbx1 in craniofacial development, we explored the possibility of an interaction between Tbx1 and Tbx2a. The use of bimolecular fluorescence complementation revealed the interaction between Tbx2a and Tbx1, thus providing support for the idea that functional interaction between different, co-expressed Tbx proteins could be a common theme across developmental processes in cell lineages and tissues. Together, this work provides mechanistic insight into the role of TBX2 in human disorders affecting the face and neck.

## Introduction

*Tbx2* belongs to the *T-box* family of transcription factors and its function has been actively studied during organogenesis and oncogenesis [1-7]. *In vitro*, TBX2 affects cell proliferation and/or survival by regulating the anti-apoptotic gene, p53 [8-10]. Tbx2 contains domains for activating and repressing gene transcription and performs these roles in a context-dependent manner [11-14]. In mice, *Tbx2* is involved in the development of several organs, including the limbs [15], heart [16], mammary gland [1] and pharyngeal arches (PA) [17]. Interestingly, the pharyngeal expression of *Tbx2* is conserved across species, including frog, chick and mice [18-22]. 

In all gnatostomes, the pharyngeal apparatus derives from a series of bulges located on the lateral surface of the head that develop into the pharyngeal arches (PAs). Cells of all three embryonic germ layers— endodermal pouches, mesenchymal condensations and neural crest cells —contribute to the formation of the PAs, choreographing their respective movements to become juxtaposed to facilitate morphogenesis based on these molecular interactions [23-25]. During this process, the anterior lateral endoderm branches into slits or out-pockets, which extend dorsoventrally to reach the ectoderm and separate the PAs. The anterior lateral endoderm also gives rise to the thyroid gland, the parathyroid gland and the thymus [23,26]. Neural crest cells (NCCs) migrate into the arch complex to develop into skeletal elements and other connective tissue structures of the PAs, whereas mesenchymal condensations form muscles [23,27-31]. 

Despite the high incidence of birth defects affecting the face and neck in humans, the genetic and molecular mechanisms of these disorders remain largely unknown [32]. One of the better described craniofacial malformations is DiGeorge’s syndrome, which is characterized by parathyroid hypoplasia, thymic hypoplasia, and outflow tract defects of the heart mostly linked to mutations in *TBX1* [33,34]. *TBX2* mutations have not been described in humans; however, the microdeletion at 17q23.1q23.2, which contains the *TBX2* locus, has been linked to a number of abnormalities, including those of the face and neck [35,36]. In addition, the *de novo* duplication within this region results in a partial overlapping complex phenotype reminiscent of DiGeorge’s syndrome [37]. 

Other members of the T-box family, such as Ntl, Spt and Tbx6, have been shown to interact with each other in co-expression domains to exert regulatory activity [38]. This is achieved via the formation of homo- or heterodimers that bind at duplicated palindromic T-box sites [39,40]. Thus, it would be informative to characterize T-box protein function in a focal domain/tissue to elucidate the respective interacting molecular network. Given the overlap in the expression of *tbx1* and *tbx2a* in the early stages of PA formation, Tbx1 and Tbx2a may form functional heterodimers. In this study, we report the role of *tbx2a* in the development of PAs in zebrafish. We demonstrate that *tbx2a* is primarily required for morphogenesis of the endodermal pouches, and subsequently affects the development of mesenchymal condensations and NCC differentiation. Our results support this idea and demonstrate that Tbx2a function is essential and non-redundant in the morphogenesis of the PAs.

## Results

### *tbx2a* is co-expressed with *tbx1* in the endoderm of the PAs

**Figure 1:**
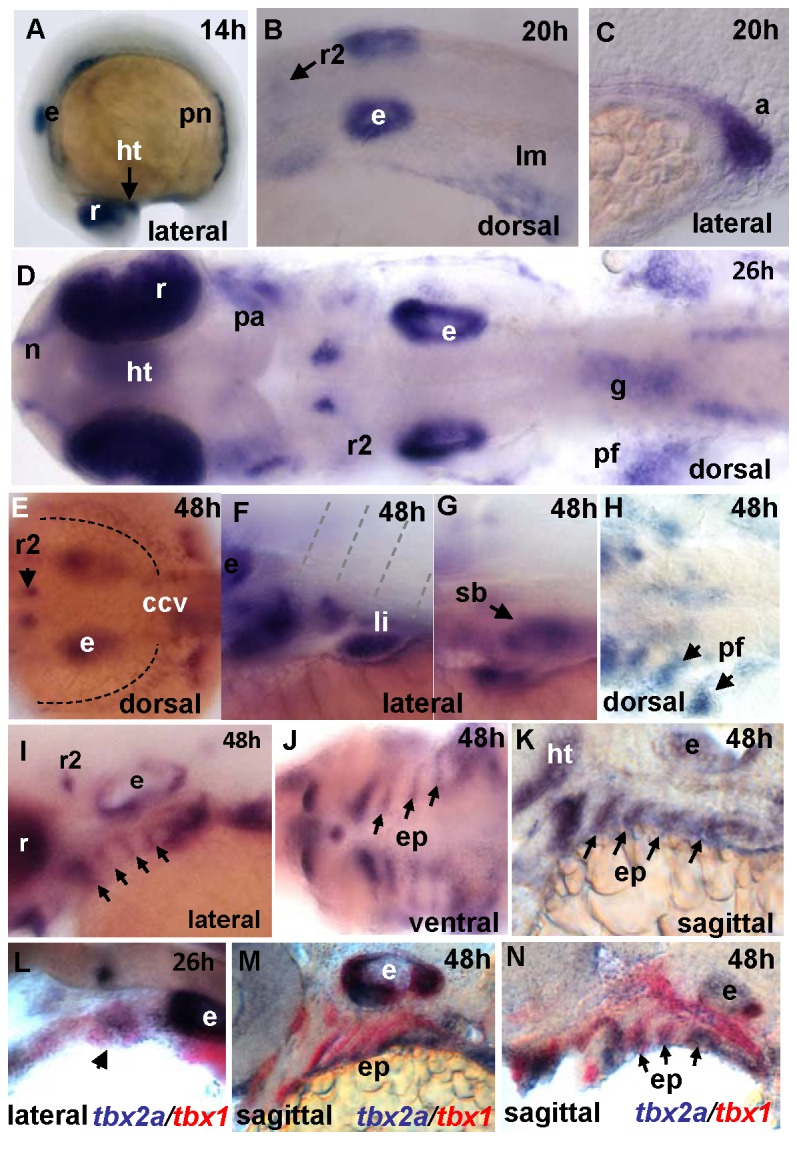
Expression pattern of *tbx2a* during development as detected by whole-mount in situ hybridization (WISH) (14-48 hpf). (**A**) Lateral view of 14 hpf embryo. Lateral-dorsal view of 20 hpf embryo (**B**) hindbrain and (**C**) lateral view of the anus. (**D**) Composite image showing a dorsal view of 26 hpf embryo. (**E**-**K**) 48 hpf embryo. (**E**) Dorsal view of the hindbrain. (**F**) Lateral view at the level of somite 2. (**G**) Lateral view of the swim bladder. (**H**) Dorsal view of the pectoral fins. (**I**-**K**) Pharyngeal arches in a (**I**) ventral view and in (**J**-**K**) sagittal sections. Two-color WISH for (K) *dlx2* (magenta) and *tbx2a* (red), (**L**-**N**) *tbx2a* (magenta) and *tbx1* (red). Abbreviations for all figures: a: anus; pa: pharyngeal arches; ccv: common cardinal vein; e: ear; ep: endodermal pouch; g: gut; r: retina; r2: rhombomere 2; ht: hypothalamus; h: hours post-fertilization; li: liver; lm: lateral mesoderm; n: nasal pits; ncc: neural crest cells; pf: pectoral fin; pn: pronephric ducts; sb: swim bladder; v: vagal nucleus.

*tbx2a* transcripts were first detected by WISH at 11 hpf; by 14 hpf, *tbx2a* transcripts were identified in the dorsal eye primordia, the otic placode and mesoderm lateral to the otic placode, the ventral diencephalon and as two lateral stripes of the intermediate mesoderm contributing to the pronephric epithelia (Figure 1A). At 20 hpf, *tbx2a* expression at low level appeared in rhombomere 2 (Figure 1B, D), and in the pronephric ducts (Figure 1C). By 24 hpf, the expression appeared as two stripes of cells proximal to the eyes (Figure 1D); these stripes will later develop into the mandibular and hyoid arch mesenchyme. *tbx2a* expression was also detected in the olfactory placode, ventral diencephalon, pectoral fin buds and anterior gut at this time point (Figure 1D). By 48 hpf, the transcript was detected in a thin layer of cells lining the yolk that will later form the common cardinal vein (Figure 1E). *tbx2a* expression was also detected in the liver (Figure 1F), the swim bladder primordium (Figure 1G ), and in the pectoral fins (Figure 1H). A close inspection of the PAs showed *tbx2a* expression in the endodermal pouches, but not in the *dlx2a*-positive NCC-derived compartment (Figure 1I, J and K). In summary, *tbx2a* is expressed in many domains that are evolutionarily conserved from teleosts to mammals according to the common ancestral origin of these organs during evolution [41].

Interestingly, the expression pattern of *tbx2a* in the PAs is reminiscent of that of *tbx1*, which shown to play a role in PA development [33]. In endodermal pouches, we identified *tbx1* transcripts co-localized with *tbx2a* transcripts (Figure 1L, M and N). However, here the *tbx1* expression domain covered the full-length of the pouches; i.e., *tbx1* expression appeared broader than that of *tbx2a*, which covered only the most ventral part of the pouches. 

### Tbx2a is required for development of the PAs

**Figure 2:**
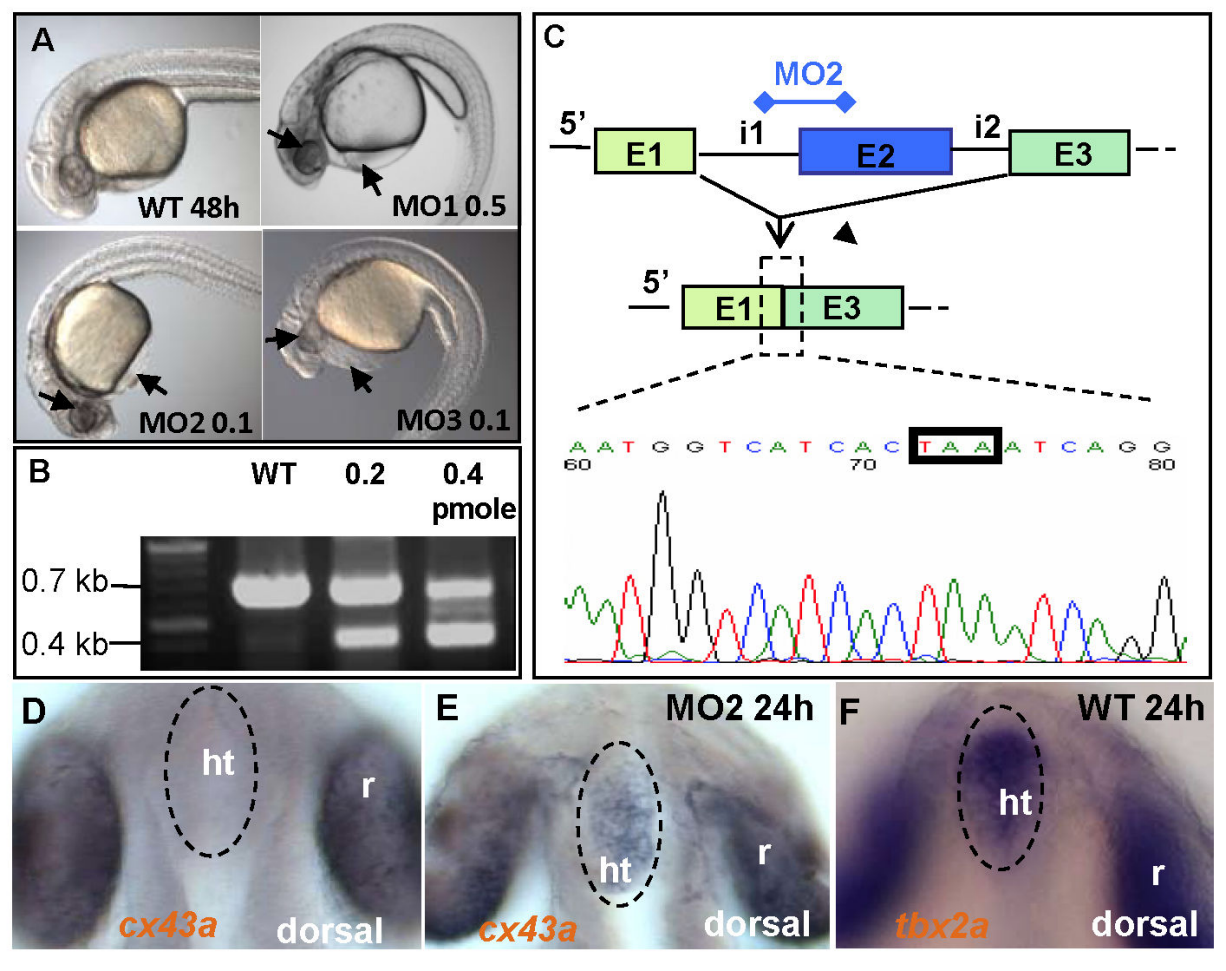
Morpholino activity. (**A**) 48 hpf live MO1/MO2/MO3-injected embryos (doses of 0.5 pmole/0.1 pmole/0.1 pmole, respectively) exhibit hydrocephalus, heart edema (arrow), body curvature, and reductions to the ears and eyes (arrow). (**B**) Amplified mature transcripts of MO2 morphants on a 0.8% agarose gel. Only one mature transcript is present in the WT, with reduction of the transcript detected in morphants injected with MO2. An additional small transcript was detected caused by the binding of MO2 to the acceptor site of intron 1 and excision of a fragment containing intron 1, exon 2 and intron 2. (**C**) Mature transcript results from joining of exon 1 and exon 3 and a frame shift-induced stop codon (TAA-black box) at the beginning of exon 3. *cx43a* expression is normally absent in the ventral diencephalon of control embryos (**D**), but is ectopically activated in the *tbx2a* morphants, (**E**) in the domain of *tbx2a* expression, and (**F**) in the ventral diencephalon.

*tbx2a* is expressed only zygotically and here we showed that its expression was detected after 10 hpf by both WISH and RT-PCR. To investigate the developmental role of *tbx2a*, we used three antisense morpholino oligonucleotides (MOs) to target different sites: MO1, which targets the splice donor site of intron 1; MO2, which targets the splice acceptor site of intron 1; and MO3, which targets the splice acceptor site of intron 5. These three MOs target the T-box domain or the transactivation domain, both of which are essential for the protein’s function [42]. All MOs worked at high efficiency and produced similar phenotypes that correlated with the expression pattern of *tbx2a*. The MO-injected embryos (morphants) survived up to 7 days post-fertilization (dpf). The developmental defects included dysmorphic PAs, small ears, malformed anus, curved body, cardiac edema, enlarged yolk sac and a failure of swim bladder inflation (Figure 2A). Of the three MOs, MO2 was the most efficient (0.1-0.2 pmole/embryo). We sequenced the aberrant transcript generated after MO2 injection and found that the resulting mRNA lacked the entire intron 2 caused by the introduction of a premature stop codon immediately preceding exon 3 (Figure 2B, C). Thus, MO2 led to the formation of a non-functional Tbx2a peptide that lacked both the DNA binding T-box and the 3’-transactivation domains [43]. It has been shown that Tbx2 transcriptionally represses *connexin 43* (*cx43*) [13,44]. In MO2 morphants, *cx43a* expression was upregulated in the ventral diencephalon, where *tbx2a* was expressed (Figure 2D–F). This suggested that MO2 efficiently down-regulated Tbx2a function. As such, MO2 was used in all subsequent experiments. 

**Figure 3:**
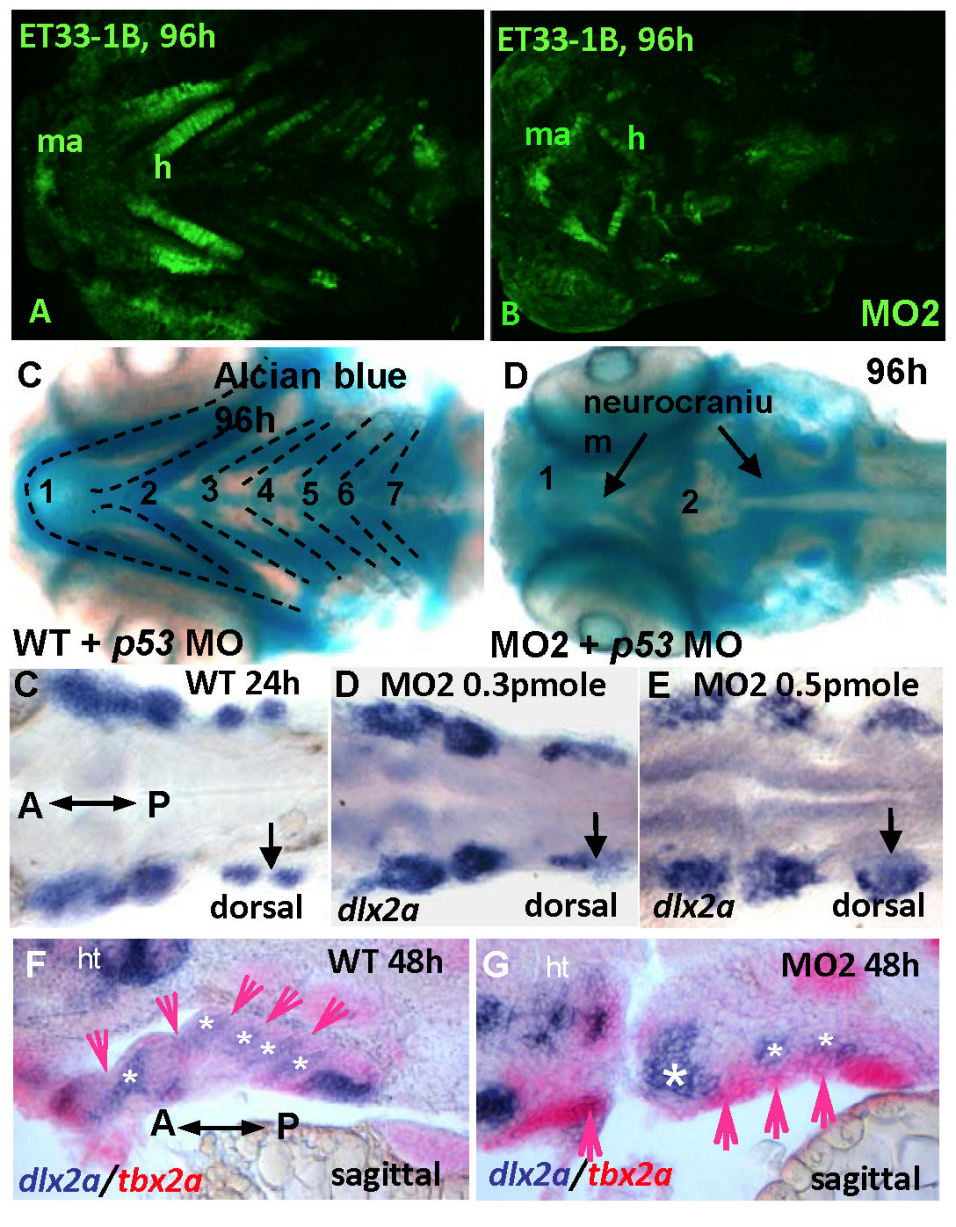
Knock-down of *tbx2a* affects pharyngeal arches. (**A**, **B**) *tbx2a* knock-down results in a loss of GFP-positive pharyngeal arches visualized on a background of ET33-1B transgenics. Alcian Blue-stained cartilages are present in the pharyngeal arches of *tp53* morphant (**C**), but not in the *tbx2a*/*tp53* morphants (**D**). At 24 hpf, *dlx2a*-positive streams of migratory NCCs (**C**) develop normally in the morphant (**D**) even after injection of a high dose of MO (**E**). At 48hpf, the patterning of posterior arches is affected (arrows and asterisks in **F, G**).

We first tested the involvement of p53 in this system. Co-injection of MO2 and p53 MO resulted in the same phenotype as that of MO2 alone; this ruled out a p53-mediated nonspecific effect of the MO (data not shown). Given the deficiency of the PAs in Tbx2a morphants, we next focused our attention on the role of Tbx2a in the organs that derive from this structure. The GFP transgenic line ET33-1B, a transposon remobilization derivative of ET33, was previously obtained in a Tol2-mediated enhancer trap screen and found to map to Chr.16: 35255049 in the intron of *me1* [45]. This line shows strong GFP expression in the PAs, cleithrum and swim bladder (Figure 3A, Figure S1). Me1 expression is under the control of thyroid hormone and plays a role during the formation of the PAs [46,47]. Thus, this transgenic line represents a sensitive tool to study PA morphogenesis *in vivo*. At 96 hpf, MO2 morphants of ET33-1B displayed disorganization of all PAs (Figure 3B), with a malformed hyoid and defective cartilage in posterior PAs (Figure 3C, D) as demonstrated using Alcian Blue staining [48]. This cartilage defect may be attributed to the undeveloped mesenchymal condensation of NCCs and mesodermal cells [23]. Since *tbx2a* is also expressed in the hindbrain, but not in the mesodermal core (Figure 1D), this patterning defect in the NCC-derived cartilage may have originated from changes in the hindbrain organization. However, we found that the expression of the hindbrain marker *hoxa2* [49] was not affected in the morphants, indicating no significant change in the hindbrain organization (Figure S2A-F). 

We next examined the migration of NCCs using the *dlx2a* probe [50]. By 24 hpf, NCCs migrated as streams of *dlx2a*-positive cells that appeared as three distinct groups in both the morphants and controls (Figure 3C-D). During development, the formation of the endodermal pouches further separates these three groups of NCCs into seven mesenchymal condensations and the double (*dlx2/tbx2a*) *in situ* staining again illustrates the fact that *tbx2a* is not expressed in the NCC (Figure 3F; [23]). However, in the morphants, this process was affected, with endodermal pouches failing to out-pocket and mesenchymal condensation becoming fused (arrows and asterisks, Figure 3G). Taken together, these results demonstrated that *tbx2a* is not involved in the patterning and migration of NCCs, but is required for proper mesenchymal condensation and subsequent cartilage differentiation.

### *tbx2a* is required for late development of endodermal pouches

**Figure 4:**
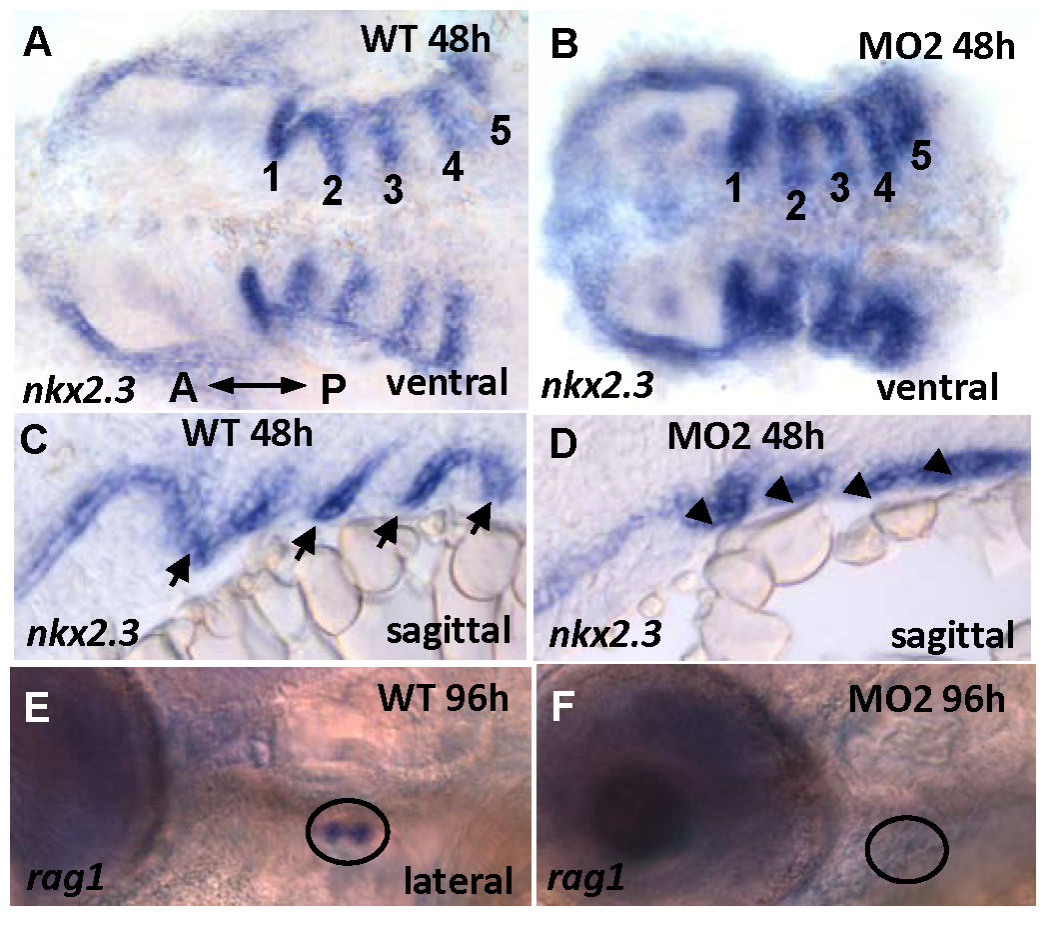
*nkx2.3* expression. *nkx2.3* is expressed in the segmented endodermal pouches in control (**A**, **C**) and morphant embryos. These segmented endodermal pouches are almost fused in the morphants (**B**, **D**). The *rag1*-positive thymus, a derivative of the endodermal pouch (**E**) is absent in the morphant (**F**). A: anterior, P: posterior.

Although *tbx2a* expression was restricted to the endodermal compartment of the arches, its knock-down affected normal formation of the entire PA; this indicated the possible master role of *tbx2a* during PA development. To evaluate this possibility, we analyzed induction of the endodermal pouches between control and morphant embryos using *nkx2.3*, a specific marker of the endodermal pouch. *nkx2*.3 is expressed in the five domains between the six PAs [51]. In a ventral view, *nkx2.3* revealed five pouches present in both the control and morphant embryos (Figure 4A, B), suggesting that induction of the endodermal pouches is not dependent on Tbx2a function. However, sagittal sections showed severely shortened *nkx2.3* expression domains in the morphants as compared to controls (Figure 4C, D), suggesting a failure of the endodermal pouch to elongate and interdigitate with the NCC-derived compartment along the proximodistal axis. This correlates well with the *tbx2a* expression observed in the ventral aspect of the pouches. The thymus primordium appears in zebrafish larvae at 54 hpf as a derivative of the caudal half of endodermal pouch 3 [52]. In controls, we identified maturing B and T lymphocytes of the thymus from 96 hpf (Figure 3E) with positive *rag1* staining [53]. However, in the morphants, the thymus was not detected (Figure 3F), further illustrating the endodermal pouch deficiency caused by down-regulation of *tbx2a*.

**Figure 5:**
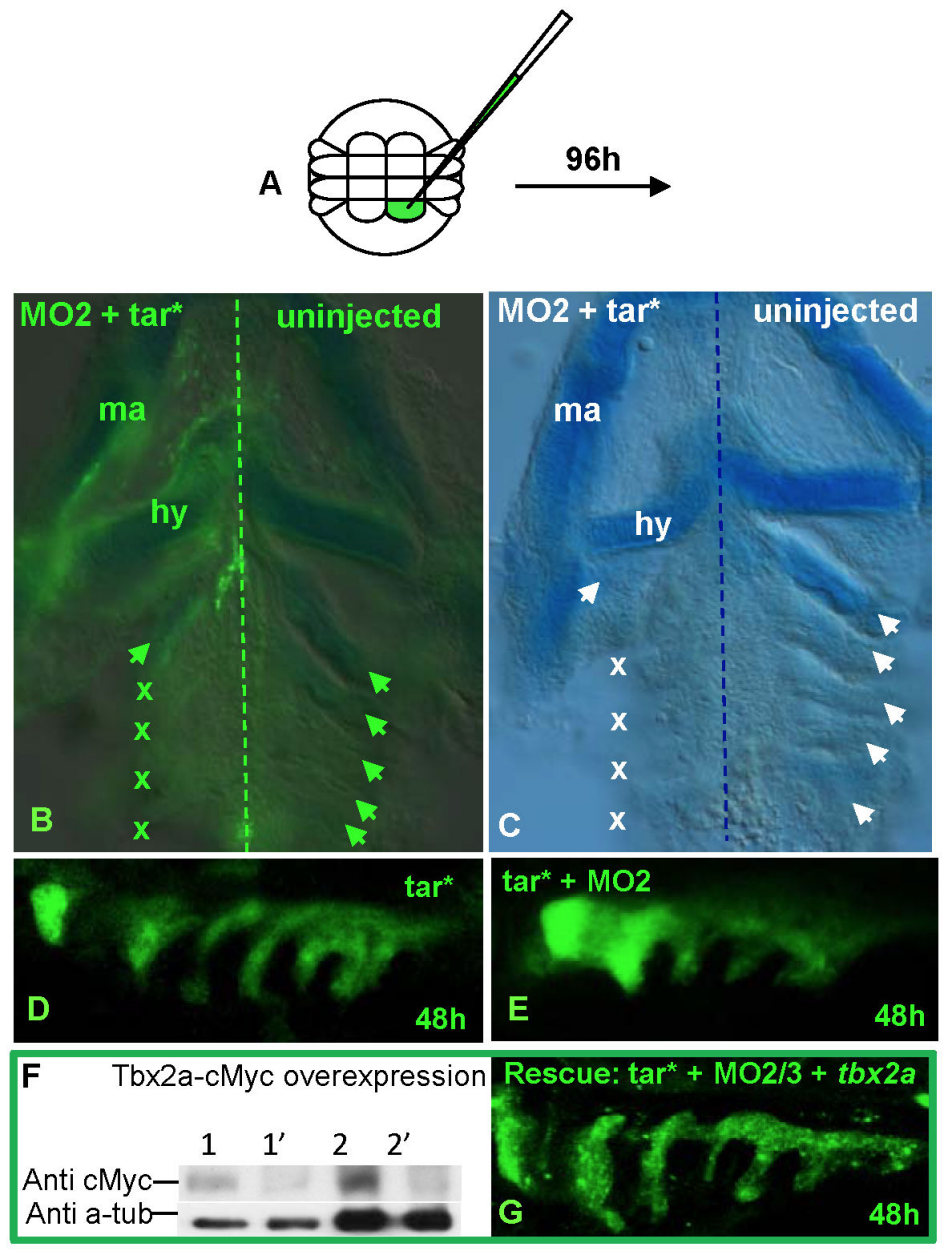
Endodermal pouch-specific knock-down of *tbx2a* causes an anomaly of the pharyngeal arches rescued by *tbx2a* mRNA. (**A**) Taram-A* (tar*) mRNA injected into the marginal blastomere at the 16-cell stage gives rise to mesendoderm, from which the endodermal pouches derive. (**B**) Co-injection with MO2 affects the posterior pharyngeal arches (crosses). (**C**) Alcian Blue staining viewed under bright field microscope. Confocal imaging of the endodermal pouches upon co-injection with fluorescent dye and tar* mRNA in the (**D**) control and (**E**) the morphants co-injected with MO2. These morphants exhibit shortened and thickened endodermal pouches. (**F**) Western blot of total lysates from *c-myc*-tagged *tbx2a* mRNA-injected embryos (lane 1, 20 µg; lane 2, 100 µg) and non-injected embryos (lane 1’, 20 µg; lane 2’, 100 µg). (**G**) Rescued MO2-injected morphants with *tbx2a* mRNA show elongated endodermal pouches.

To demonstrate that the endodermal pouches are essential for the morphogenesis of an entire arch complex, we knocked down *tbx2a* expression selectively in the endodermal pouches. Previous studies have shown that injection of mRNA encoding the constitutively active Taram-A (Taram-A*) type I subunit of the TGF-β receptor [54] into a 16-cell stage single blastomere can direct progenitors of the marginal blastomere to develop as anterior endodermal derivatives [55]. We found that embryos co-injected with 0.6 pg mRNA of Taram-A* and 70 kD fluorescein dextran developed normally with unilaterally labeled endodermal pouches of the PAs (Figure 5A). However, in the experimental embryos, the further addition of 0.05 pmole MO2 severely affected the cartilage of the posterior arches on the injected side, as observed with Alcian Blue staining (Figure 5B, C). This phenotype was partially rescued by adding 10-15 pg of *tbx2a* mRNA into the injection mix. In this case, about 30% of injected embryos developed discrete pouches extending along the full length of the dorsoventral axis (Figure 5D, E, G; n = 10/32). These results are significant in view of the dose-sensitive effects of the T-box proteins [56]. 

### Tbx2 interacts with Tbx1

**Figure 6:**
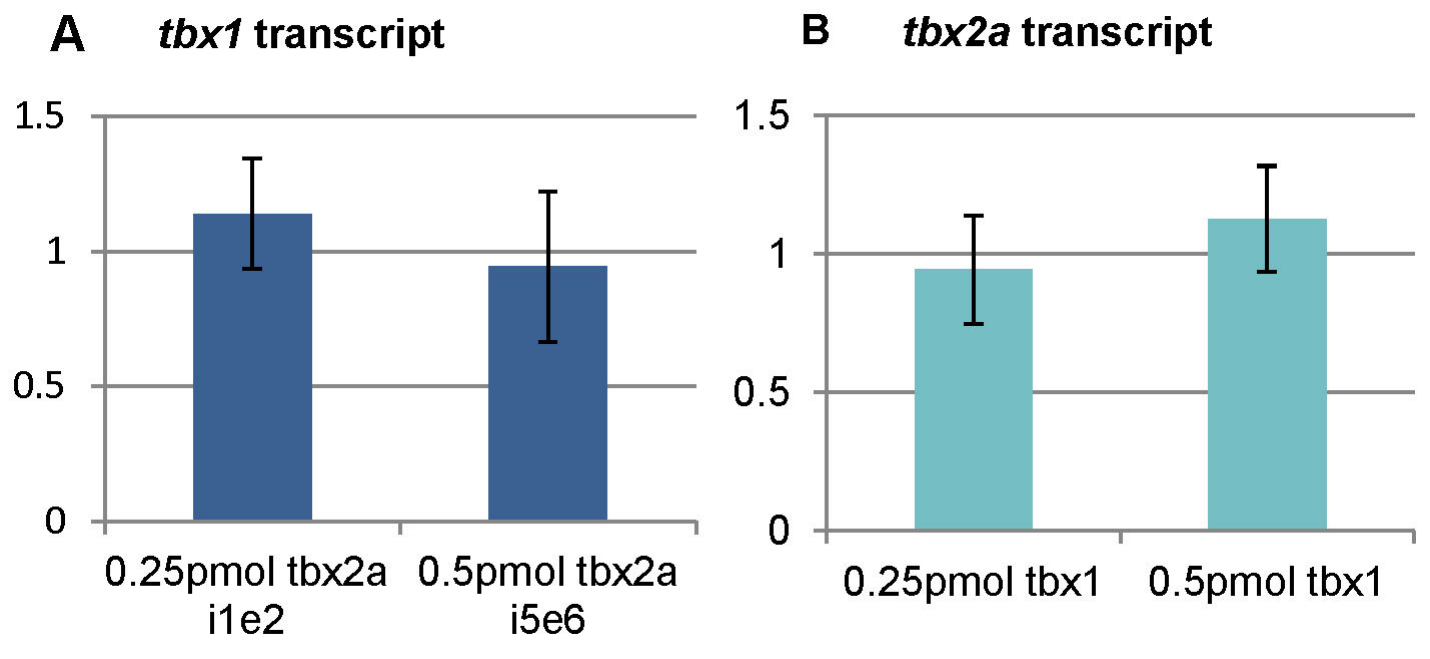
Tbx2a and Tbx1 do not regulate expression of each other. Fold-change of the expression level of Tbx2a and Tbx1 relative to the control (1x change) at 30 hpf. (**A**) *tbx2a* MO2 and MO3 had no significant effect on the expression of *tbx1*. (**B**) *tbx1* MO had no significant effect on the expression of *tbx2a*.

Given the expression patterns of *tbx1* and *tbx2a* and the phenotypes of embryos deficient in Tbx1 and Tbx2a are rather similar ([33]; this paper), we proposed that these proteins interact during PA development. The expression levels of *tbx1* and *tbx2a* were measured after reciprocal knock-down. RT-PCR did not detect obvious changes in the expression of *tbx1*, when *tbx2a* was knocked down using different doses of MOs and *vice versa* (Figure 6A, B) suggesting that neither one gene regulates the other.

**Figure 7:**
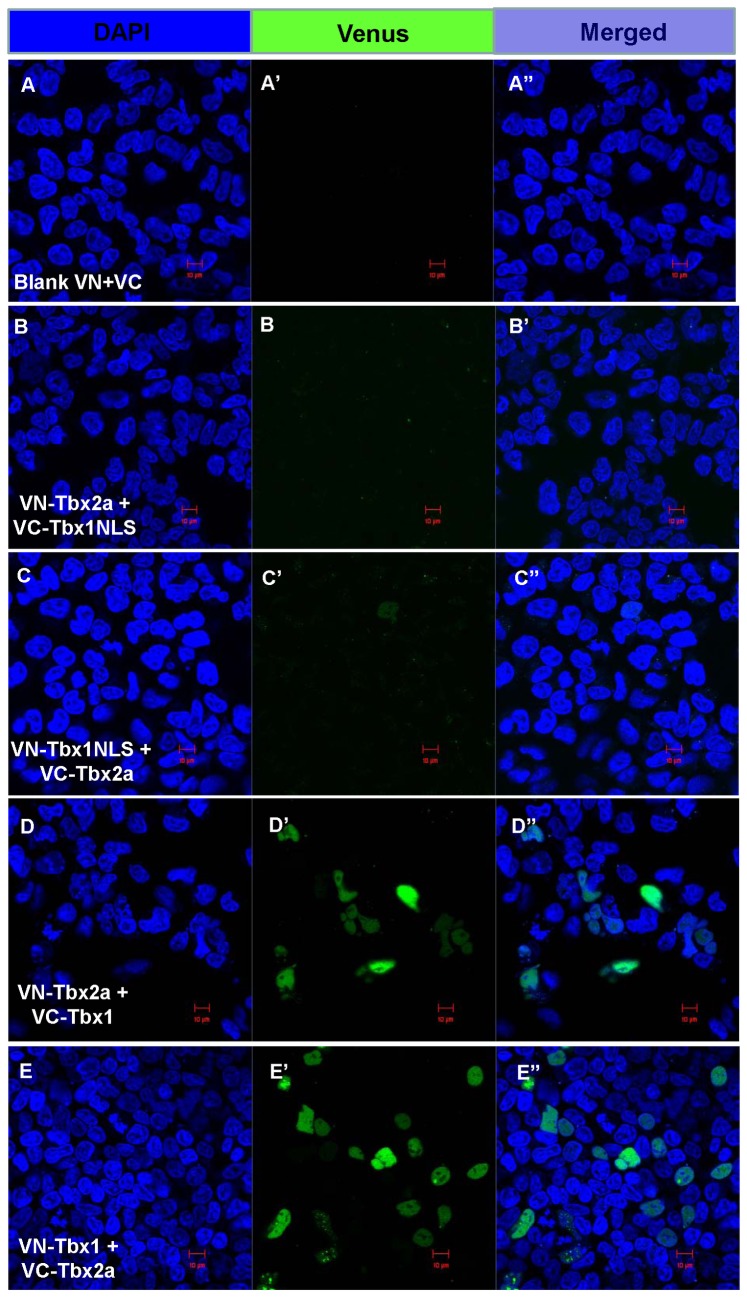
Cloned-Tbx1 and Tbx2a in Venus constructs co-localized in the nucleus of transfected 293T cells. Cells were co-transfected with 0.5 µg blank Venus constructs - VN and VC, only a few cells fluoresced in cytoplasm and nucleus (**A, A**’**, A**”**)**. A few cells amongst those co-transfected with 0.5 µg VN-Tbx2a and VC-Tbx1NLS (**B, B**’**, B**”) or 0.5 µg VN-Tbx1NLS and VC-Tbx2a (**C, C**’**, C**”) fluoresced weakly. In contrast, cells nuclei co-transfected with 0.5 µg VN-Tbx2a and VC-Tbx1 (**D, D**’**, D**”) or 0.5 µg VN-Tbx1 and VC-Tbx2a (**E, E**’**, E**”) fluoresced in many cells. Statistical data are shown in Table S1.

It is known that T-box proteins bind to the T-domain palindrome DNA sequence as dimers [57]. We asked whether Tbx2a and Tbx1 interact upon binding to DNA during development of PA. To detect an interaction between Tbx2a and Tbx1, the BiFC system [58] was adapted. *tbx1* and *tbx2a* were N-terminal tagged by VN154m10 and VC155 to produce DNA constructs encoding fusion proteins of VN-Tbx1, VC-Tbx1, VN-Tbx2a and VC-Tbx2a. These experiments were performed in HEK 293T cells. The HEK 293T cells were plated (2x10^5^) and then transfected with 0.2-0.4 µg of DNA of each construct in pairs. In controls with blank VN and VC constructs, fluorescent cells were extremely rare (several cells per 3.5 cm plate) with both nuclear and cytoplasmic fluorescence, which likely resulted from aberrant excessive transfection (Figure 7A, A’, A”). The constructs containing a small fragment of Tbx1 C-terminus (385 to 451) containing nuclear localization signal [59] tagging VN/VC served as negative controls (Figure 7B, B’, B” and 7C, C’, C”). These are VN-Tbx1NLS and VC-Tbx1NLS that localize to the nucleus (data not shown), but unable to interact directly with Tbx2a. In these control experiments we observed a background nuclear fluorescence, which was significantly lower than that in the experimental sets. Indeed, obvious nuclear fluorescence was detected only after transfecting a combination of VN-Tbx2a and VC-Tbx1 (Figure 7D, D’, D”) or VN-Tbx1 and VC-Tbx2a (Figure 7E, E’, E”; Table S1). These results suggested that during PA development Tbx2a and Tbx1 might interact upon binding to DNA. 

## Discussion

### *tbx2a* affects the development of the PAs by regulating endodermal pouch specification

*Tbx2* expression in the PAs is conserved across species suggesting the importance of this gene during craniofacial development. We report in this study that *tbx2a* is involved in the late morphogenesis of endodermal pouches. In the absence of Tbx2a function, endodermal pouches failed to elongate along the dorsoventral axis towards the epidermal surface. Therefore, although *tbx2a* is not involved in endodermal segmentation, it is required for pouch outgrowth. It is known that F-actin accumulation at the apical surfaces of cells in the pouch is necessary to direct and constrain the movement of endodermal cells into a narrow group with a slit-like shape [60,61]. Because N-cadherin connects to the actin cables, it may also be involved in the regulation of pouch morphogenesis. T-box factors have been shown to regulate cell adhesion molecules (e.g., *cx43*) and play roles in cell attachment and migration [12,62]. A remodeling of cell adhesion dynamics might be a constitutive part of the *tbx2a*-dependent mechanism that regulates morphogenesis of the PA endoderm. Development of the endodermal pouch has a leading regulatory role in the development of the PAs [23-25,61,63], and our data provide strong support for the role of *tbx2a* in this aspect of PA formation. The effect of *tbx2a* on other cell lineages is indirect and could be due to disturbances in the regulatory interactions between endodermal pouches and other cells contributing to PA development, which probably take place downstream of Tbx2a. 

Although *Tbx2* is expressed in the PA in mice, its specific function in this organ is not well documented. In chick, *Tbx2* is expressed in both the PA epithelium and mesenchyme. In contrast, two zebrafish paralogues, *tbx2a* and *tbx2b*, are expressed in the PAs in a complementary manner; i.e., while the expression of *tbx2a* is restricted to the endodermal pouches, *tbx2b* is expressed in the arch mesenchyme (data not shown). This split in expression probably results in discrete functions of these two paralogues. Elucidating their respective roles in each compartment may reveal critical interactions between the various compartments of the arch during PA development. 

*Tbx2* and *Tbx3* often co-express and act redundantly in their overlapping domains [64,65]. However, zebrafish *tbx3* is not expressed in the endodermal pouches ([66]; data not shown). In mice, a Tbx2-null mutation results in hypoplastic PAs [17], while double mutations in Tbx2 and Tbx3 cause severe defects in the PAs accompanied by improper segmentation [67]. However, in this study, we show that Tbx2a ablation alone is sufficient to disturb the specification of the endodermal pouches in zebrafish. 

The endodermal pouches regulate cartilage development during PA formation. Here, Tbx2a may interact with some other Tbx proteins. The expression pattern of Tbx2a is similar to that of Tbx1, and the loss of function after Tbx2a knock-down is rather similar to that observed in *vgo* mutants that are deficient in Tbx1 [33]. In this study, we provide for the first time molecular evidence of heterodimerization between Tbx1 and Tbx2a. The data suggest that the Tbx1/Tbx2a heterodimer could be an essential regulatory component in the development of the zebrafish endodermal pouches. This opens up the possibility that regulatory T-box heterodimers may be common components of the developmental mechanism acting in parallel with T-box homodimers.

Interestingly, craniofacial abnormalities reminiscent of the Tbx1-linked DiGeorge’s syndrome have been observed in patients with a microdeletion at 17q21-22, leading to TBX2-haploinsufficiency [35,36]. The partial duplication of this region resulted in a complex phenotype similar to that seen in patients with DiGeorge’s syndrome [37]. Recently, Tbx1, Tbx2 and Tbx3 have been proposed to form an interacting network where Tbx2/3 act as modifiers, and it has been suggested that a deficiency in either Tbx2 or Tbx3 could result in the development of a phenotype reminiscent of the cardio-pharyngeal phenotype found in TBX1-haploinsufficient 22q11.2DS patients [67]. In zebrafish, we show that the functions of Tbx1 and Tbx2a are non-redundant, since the loss of either Tbx1 or Tbx2a produced PA defects with a similar degree of severity. 

Our study reveals a role for *tbx2a* during the development of the endodermal component of the PAs. This functional analysis has shown for the first time that *tbx2a* is indispensable for morphogenesis, but not induction, of the pharyngeal endodermal pouches. These defects of the endodermal pouches in turn affect proper mesenchymal condensations. Whereas NCC induction and migration are initially independent of the activity of Tbx2a, their late differentiation into cartilage depends on Tbx2a function. Importantly, our data suggest that Tbx2a may interact with Tbx1 to co-regulate the development of the PAs and this process plays a role during craniofacial development. Further studies to support this hypothesis are warranted. 

## Materials and Methods

### Fish maintenance

The experiments using the wild type AB and the transgenic line SqET33-1B [45] zebrafish (*Danio rerio*) were performed according to the regulations of the Fish Facility (IMCB, Singapore) approved by the Institutional Animal Care and Use Committee (IACUC) rules (Biopolis IACUC approval #090430). The embryos were maintained at 28.5°C and staged in hours post fertilization (hpf). Pigment formation was inhibited with the use of 0.003% 1-phenyl-2-thiourea (PTU, 0.2 mM) from 22 hpf [68]. 

### Cloning of *tbx2a* gene

Total RNA was isolated using RNeasy^®^ Mini Kit (Qiagen, Hilden, Germany). RT-PCR was performed with Qiagen^®^ OneStep RT-PCR Kit. Full-length *tbx2a* was obtained with primers (Forward) 5’-GCTATGGCTTATCACCCTTTTC-3’ and (Reverse) 5’-GAAGTTTTGCGCTTTATGTCACA-3’, based on the sequence in ENSEMBLE (http://www.ensembl.org, transcript ID ENSDART00000024207). This DNA was cloned into pGEM^®^ T Easy vector (Promega, Madison, WI) for antisense RNA probe synthesis or subcloned into pCMV-Tag 5A vector (Stratagene, USA) for sense mRNA synthesis. ImageJ (NIH, Bethesda, MA) was used to estimate dot intensity for DNA bands on agarose gel images. Motif searches were performed with MyHits© 2003-2009 (http://myhits.isb-sib.ch/cgi-bin/clustalw).

### Molecular applications

mRNA synthesis was carried out according to standard procedures using the mMESSAGE mMACHINE^®^ Kit (Ambion, Austin, TX). Antisense RNA labeled with fluorescein-12-UTP (FITC) or digoxigenin-11-UTP (DIG) was synthesized *in vitro* using the MEGAscript^®^ Kit (Ambion). Protein lysates were prepared from de-yolked 1 dpf embryos (by pipetting embryos through a 1 ml tip in PBS) and were probed with monoclonal anti c-Myc (9E10, Santa Cruz, USA) or anti-α-Tubulin (Sigma-Aldrich, St. Louis, MO) antibodies. 

### Morpholino (MO)

MOs from Gene Tools LLC (Philomath, OR) were used as follows: (1) MO1 [5’-AGACCTTACCTTCCTGATTTAGTGA-3’], 0.4 pmole/embryo (targeting donor site at intron 1 of *tbx2a*); (2) MO2 [GGAAACATTCTCCTATGGACGAAAG], 0.1-0.2 pmole/embryo (targeting the acceptor site at intron 1 of *tbx2a*); (3) Mismatched morpholino-Mis-MO2 [5’-cGAAACAcTCgCCTAcGGACcAAAG-3’] (lower case denotes replaced nucleotides), 0.4 pmole/embryo (negative control for MO2); (4) MO3 [5’-TTGTCTTCTGGAAAAACAAATGTTA-3’], 0.1 pmole/embryo; (5) tbx1-MO [5′-GAT GTCTCCAATAGATAATGTGTCG-3′], 0.1 pmole/embryo (targeting 5’UTR of *tbx1*) [69]; (6) p53-MO [5’- GCGCCATTGCTTTGCAAGAATTG-3’] [70], 0.3 pmole/embryo (targeting ATG of *tp53*).

### 16-cell stage injection

Zebrafish embryos were manually dechorionated at the 1- to 4-cell stage and placed on agar molds (2% agarose in egg water). Single marginal blastomeres of 16-cell stage embryos were injected with no more than 200 pl of reagents mixed with 70 kD fluorescein dextran as a tracer. 

### Alcian Blue cartilage staining

This protocol was adopted and modified from [71]. Proteinase K-treated embryos (72 to 96 hpf) were stained for 4 h in 0.1% Alcian blue dissolved in acidic ethanol (70% ethanol, 5% concentrated HCl). Stained embryos were then washed in acidic ethanol, re-hydrated and stored in PBS containing 50% glycerol before photography. 

### Whole-mount *in situ* hybridization (WISH) and immunohistochemistry

These assays were carried out as previously described [72]. *In situ* signal for alkaline phosphatase was detected with NBT-BCIP (Sigma-Aldrich) or fast red tablets (Roche Biochemicals, Basel, Switzerland). Two-color WISH was performed using two probes labeled with DIG or Fluorescein (1.5:1 Fluorescein to DIG) for the detection of different genes. All the in situ staining was done using 20-30 embryos/set and conclusions were drawn from a phenotype prevalent in 70-90% of embryos.

### Imaging

Photography was performed on the AX-70 (Olympus, Tokyo, Japan) and Axiophot2 (Carl Zeiss Inc., Oberkochen, Germany) compound microscopes. Fluorescein-labeled specimens were visualized with the Leica MZ FLIII stereomicroscope (Leica Microsystems, Wetzlar, Germany) equipped for UV epifluorescence viewing. Confocal images were acquired using the Zeiss LSM510 scanning laser microscope (Carl Zeiss Inc.). Raw image collection and processing were performed using the LSM510 Software (Carl Zeiss Inc.). Images were processed with Adobe® Photoshop CS4 (Adobe Systems, Systems, San Jose, CA).

### Bimolecular fluorescence complementation (BiFC)

BiFC was adapted from [58]. The full-length sequence of *tbx2a* or *tbx1* or a fragment encoding the Tbx1 C-terminal peptide that encompass amino acid 385 to 451 (Tbx1NLS) were sub-cloned into the 3’-terminal of VN154m10 (carrying mutations L46F/L64F) or VC155 constructs at the pCS2 plasmid that upon expression results in the N-terminal tagged versions of Tbx proteins. Combinations of DNA constructs were transfected into HEK 293T cells using the jetPRIME transfection reagent (Polyplus, France) at the doses of 0.3-0.5 µg per construct per 3.5 cm plate. Transfected cells were stained with vital DAPI, which was replaced with DMEM medium for observation and imaging using confocal microscopy.

## Supporting Information

Figure S1 **Tbx2a KD by other two MO.** Alcian Blue staining of (**A**, **B**) MO1 morphant; (**C**) mis-MO2 control; (**D**) MO2; and (**E**-**H**) MO3 morphants.
(TIFF)

Figure S2 **Knock-down of *tbx2a* does not affect early hindbrain patterning.** (**A**-**D**) *hoxa2* expressed in rhombomeres 2 to 5 and streams of neural crest cells (NCCs) (arrows) in both WT and morphants. (**E**, **F**) *hox2a*-positive NCCs arrive at the pharyngeal region.
(TIFF)

Table S1 **Nuclear Venus-positive cells were counted under 40x objective with UV.** The visual field (viewing) was picked randomly and about 60-100 cells were evaluated in each field. Number of positive cells was noted for each viewing. Total of 15 viewings were made for each pair of constructs.
(DOCX)
