# Supplementary figures and images for: tbx2a Is Required for Specification of Endodermal Pouches during Development of the Pharyngeal Arches
Source: PLoS One. 2013 Oct 10;8(10):e77171. doi: 10.1371/journal.pone.0077171 (PMC3795029; doi:10.1371/journal.pone.0077171)

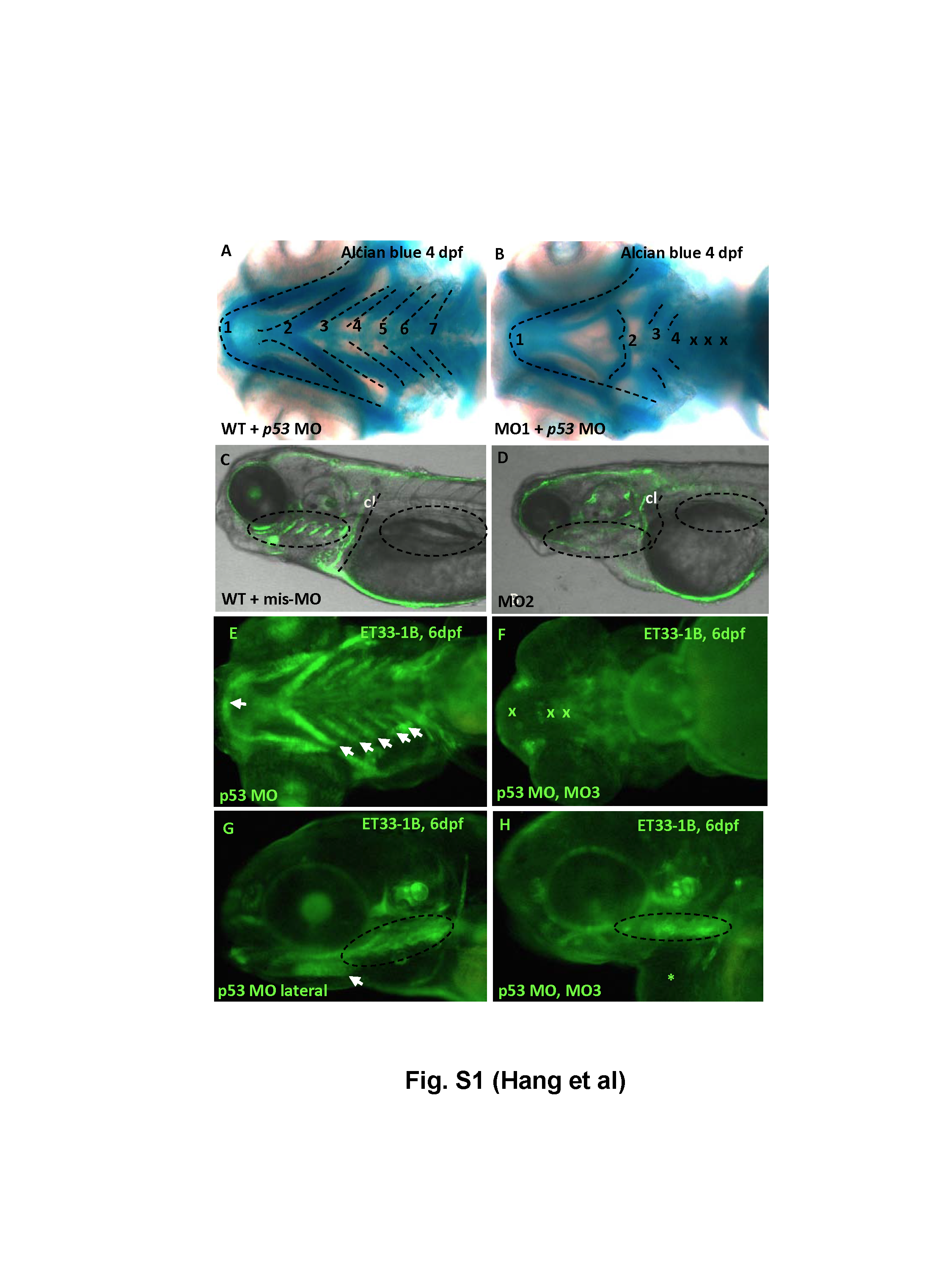

Supplement: Figure S1 — Tbx2a KD by other two MO. Alcian Blue staining of (A, B) MO1 morphant; (C) mis-MO2 control; (D) MO2; and (E-H) MO3 morphants. (TIFF) [file pone.0077171.s001.tiff]

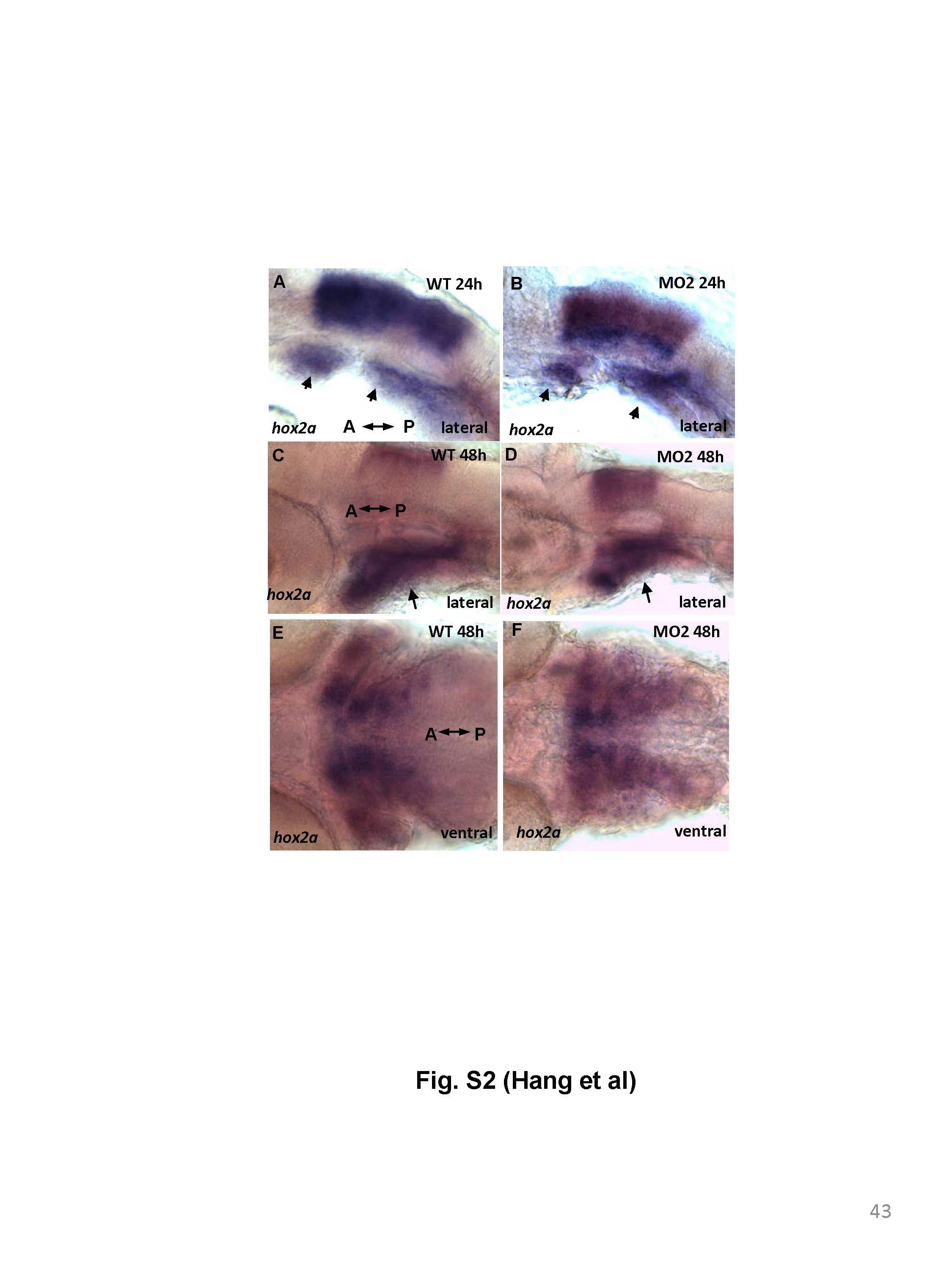

Supplement: Figure S2 — Knock-down of tbx2a does not affect early hindbrain patterning. (A-D) hoxa2 expressed in rhombomeres 2 to 5 and streams of neural crest cells (NCCs) (arrows) in both WT and morphants. (E, F) hox2a-positive NCCs arrive at the pharyngeal region. (TIFF) [file pone.0077171.s002.tiff]
