# Supplementary material for: tbx2a Is Required for Specification of Endodermal Pouches during Development of the Pharyngeal Arches
Source: PLoS One. 2013 Oct 10;8(10):e77171. doi: 10.1371/journal.pone.0077171 (PMC3795029; doi:10.1371/journal.pone.0077171)
Supplement: Table S1 — Nuclear Venus-positive cells were counted under 40x objective with UV. The visual field (viewing) was picked randomly and about 60-100 cells were evaluated in each field. Number of positive cells was noted for each viewing. Total of 15 viewings were made for each pair of constructs. (DOCX) [file pone.0077171.s003.docx]

| Venus-tagged  Constructs | Transfected  Plates | Number of random viewings/plate  (with 40x objective) | Average number of  nuclear Venus - positive cells / viewing | |
| --- | --- | --- | --- | --- |
| BlankVN/VC | 1 | 5 | 0 | |
| VN-Tbx2a/VC-Tbx1-NLS | 3 | 5 | 0-2 | |
| VN-Tbx1NLS/VC-Tbx2a | 3 | 5 | 0-2 | |
| VN -Tbx2a/VC-Tbx1 | 3 | 5 | Plate 1/1 | 12 |
|  |  |  | 1/2 | 17 |
|  |  |  | 1/3 | 9 |
|  |  |  | 1/4 | 12 |
|  |  |  | 1/5 | 9 |
|  |  |  | Plate 2/1 | 11 |
|  |  |  | 2/2 | 16 |
|  |  |  | 2/3 | 8 |
|  |  |  | 2/4 | 16 |
|  |  |  | 2/5 | 13 |
|  |  |  | Plate 3/1 | 14 |
|  |  |  | 3/2 | 12 |
|  |  |  | 3/3 | 9 |
|  |  |  | 3/4 | 15 |
|  |  |  | 3/5 | 9 |
|  |  |  | *Average* | 12 (±4) |
| VN-Tbx1/VC-Tbx2a | 3 | 5 | Plate 1/1 | 15 |
|  |  |  | 2 | 13 |
|  |  |  | 3 | 18 |
|  |  |  | 4 | 9 |
|  |  |  | 5 | 18 |
|  |  |  | Plate 2/1 | 20 |
|  |  |  | 2 | 17 |
|  |  |  | 3 | 12 |
|  |  |  | 4 | 10 |
|  |  |  | 5 | 15 |
|  |  |  | Plate 3/1 | 13 |
|  |  |  | 2 | 18 |
|  |  |  | 3 | 11 |
|  |  |  | 4 | 17 |
|  |  |  | 5 | 14 |
|  |  |  | *Average* | 15 (±5) |

**Table S1**
